# Dogslife: A web-based longitudinal study of Labrador Retriever health in the UK

**DOI:** 10.1186/1746-6148-9-13

**Published:** 2013-01-18

**Authors:** Dylan N Clements, Ian G Handel, Erica Rose, Damon Querry, Carys A Pugh, William ER Ollier, Kenton L Morgan, Lorna J Kennedy, Jeffery Sampson, Kim M Summers, B Mark C de Bronsvoort

**Affiliations:** 1 Royal (Dick) School of Veterinary Studies, University of Edinburgh, Roslin EH25 9RG, Scotland; 2 The Roslin Institute, University of Edinburgh, Roslin EH25 9RG, Scotland; 3 Centre for Integrated Genomic Medical Research, University of Manchester, Manchester, M13 9PT, UK; 4 Faculty of Veterinary Science, University of Liverpool, Neston, CH64 7TE, UK; 5 The Kennel Club, 1-5 Clarges Street, Piccadilly, London, W1J 8AB, UK

## Abstract

**Background:**

Dogslife is the first large-scale internet-based longitudinal study of canine health. The study has been designed to examine how environmental and genetic factors influence the health and development of a birth cohort of UK-based pedigree Labrador Retrievers.

**Results:**

In the first 12 months of the study 1,407 Kennel Club (KC) registered eligible dogs were recruited, at a mean age of 119 days of age (SD 69 days, range 3 days – 504 days). Recruitment rates varied depending upon the study team’s ability to contact owners. Where owners authorised the provision of contact details 8.4% of dogs were recruited compared to 1.3% where no direct contact was possible. The proportion of dogs recruited was higher for owners who transferred the registration of their puppy from the breeder to themselves with the KC, and for owners who were sent an e-mail or postcard requesting participation in the project. Compliance with monthly updates was highly variable. For the 280 dogs that were aged 400 days or more on the 30^th^ June 2011, we estimated between 39% and 45% of owners were still actively involved in the project. Initial evaluation suggests that the cohort is representative of the general population of the KC registered Labrador Retrievers eligible to enrol with the project. Clinical signs of illnesses were reported in 44.3% of Labrador Retrievers registered with Dogslife (median age of first illness 138 days), although only 44.1% of these resulted in a veterinary presentation (median age 316 days).

**Conclusions:**

The web-based platform has enabled the recruitment of a representative population of KC registered Labrador Retrievers, providing the first large-scale longitudinal population-based study of dog health. The use of multiple different methods (e-mail, post and telephone) of contact with dog owners was essential to maximise recruitment and retention of the cohort.

## Background

To date there have been no longitudinal population-based epidemiological studies that estimate the incidence and/or prevalence of canine diseases. Representative information on the pattern of disease in the general canine population is impossible to obtain from data studies utilizing secondary (referral) centres, because of the well-recognised “referral bias” [1], owing to variables including geographical location and wealth of owners. Studies evaluating presentations to primary care practices alone [2] are rare. Furthermore the prevalence of clinical signs or illnesses in dogs which are not subsequently subjected to veterinary presentation has, to our knowledge, never been reported. The paucity of published data in this field is striking.

Reports of breed associated disease risks are either anecdotal, based on referral [3] or on insurance data [4] and are thus subject to sampling bias. Meta-analysis of the available information on breed-specific genetic diseases suggests that they have inadvertently arisen as a consequence of selection for breed standards or by chance [5,6]. However most of the previous studies reported have been retrospective or cross-sectional, and did not capture longitudinal data regarding the clinical, lifestyle, environment, diet or reproductive history of individuals. Thus it is not surprising that non-genetic influences on common canine diseases remain poorly characterised.

Longitudinal studies of health are the most powerful mechanism for determining environmental and lifestyle influences on the development of disease [7]. In comparison to cross-sectional studies, longitudinal studies avoid recall bias, enable sampling at appropriate time points and allow temporal association between risk factors and disease to be established and changes of phenotype with age to be identified. Studies of human birth cohorts, such as the Avon Longitudinal Study of Parents and Children [8], have revealed many unsuspected environmental and genetic risks and associations for a wide range of human phenotypes, such as obesity [9] and cognitive functions [10]. The scale of such studies is ever increasing, with newer studies such as the 2012 Birth Cohort Study which will track the growth, development, health, well-being and social circumstances of over 100,000 UK children from gestation through the early years of their childhood. Similarly the UK Biobank project is collecting historical and prospective health data, biological samples and physiological measurements from over 500,000 participants aged 50–65 [11]. No such large-scale studies have ever been attempted using a general dog population, although focused lifetime birth cohort studies of other species such as lambs [12,13] and cattle [14] have been reported.

The internet potentially offers a rapid and efficient method for capturing data on health, wellbeing and lifestyle, and has been used to record information about pet ownership [15]. Internet-based data capture and recording systems have been widely used to record human health data and provide a cost effective and simple method for obtaining epidemiological information [16,17]. To date the internet has been used in veterinary medicine to capture end of life information [18]. Internet-based recording systems are particularly attractive for assembling longitudinal data, since participants can submit data at their own convenience, as well as allowing the incorporation of inexpensive and unobtrusive electronic reminder systems into the study design and such systems are highly scalable. In 2009, 76% of household in the UK had access to the internet [19].

This paper describes the design and recruitment for the first large scale (national) longitudinal study of canine health, called “Dogslife”. The study is collecting information on the health and well-being of a cohort of Labrador Retrievers over their first few years of life. The ultimate goal of the study is to identify environmental and genetic risk factors for the development of canine diseases, and subsequently inform future risk reduction strategies.

## Methods

The study was approved by the Veterinary Ethical Review Committee of the University of Edinburgh.

### Study population

The study population was Kennel Club (KC) registered Labrador Retrievers born on or after 1^st^ January 2010 and present in the United Kingdom (UK) at the time of registration. Puppies are registered with the KC after birth by the breeder. The breeder can keep their puppy, or transfer the puppy to a new owner, which typically occurs at around the time of their first routine vaccination between six and eight weeks of age. The new owner can transfer the KC registration of their puppy to themselves at any time after they obtain their puppy, but they are not obliged to do so.

### Recruitment

Recruitment of dogs started with the launch of the website on the 1^st^ July 2010. To maximise early recruitment all dogs born after 1^st^ January 2010 were considered eligible for recruitment. The KC reported all new Labrador Retriever registrations (puppies registered at birth by the breeder) and transfers of registration (dogs moving to new homes from the breeder) to the Dogslife project. All KC registrations from 1st January to 30^th^ June 2010 were provided as a single file but from 1^st^ July 2010, they were provided on a daily basis through electronic data transfer directly into the Dogslife database. Registration data included a unique KC number, date of birth, names of the sire and dam, KC name, gender and colour. The KC number was used as the unique identifier of each dog. For all transfers of registration, the new owners were asked by the KC to give consent to their name, e-mail address and postal address being forwarded to third parties at the time of registration with the KC (41% consenting to contact by e-mail and 54% consenting to contact by post, resulting in 61% consenting to the use of one and/or the other).

**Figure 1:**
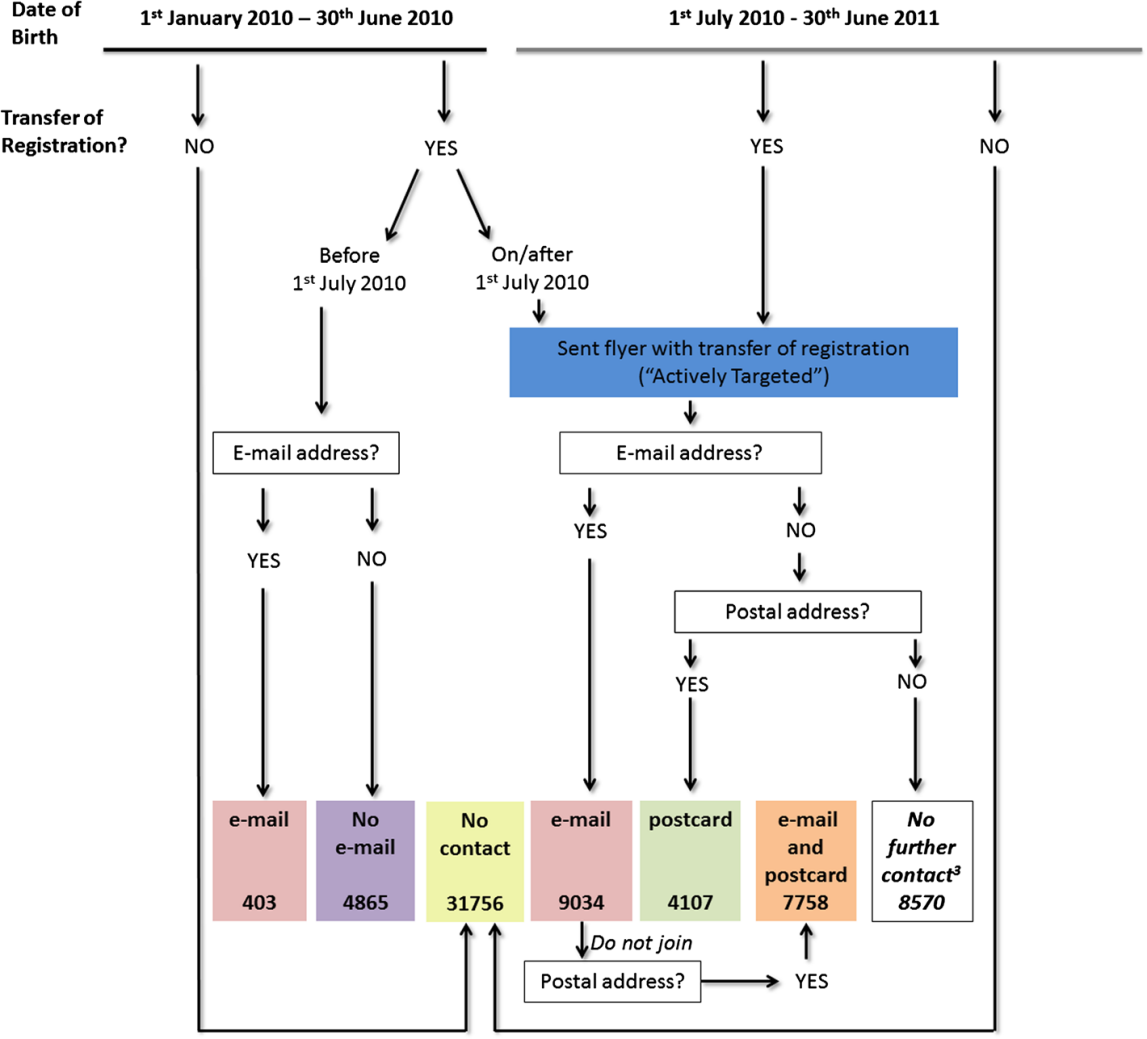
**Description of the study population and recruitment method. **All KC registered Labrador Retriever dogs born on or after 1^st ^January 2010 were eligible to join the project. The project launched on 1^st ^July 2010 and “actively targeted” dogs whose registration was transferred on or after this date.

Where transfer of registration occurred before 1^st^ July 2010 the new owners were invited to join the project by e-mail once on the 12^th^ July 2010 (Figure 1). Where transfer of registration occurred on or after 1^st^ July 2010 recruitment was more “active”. New dog owners were sent a black and white A5 sized flyer with their transfer of registration documentation inviting them to join the project. For those who consented to their contact details being used, this was followed by an automated e-mail invitation to participate if they had not joined Dogslife within seven days of the transfer, and a postal invitation sent out on a brightly coloured postcard seven days later if they still had not joined (Figure 1). Each eligible participant was only sent one e-mail and / or postcard.

**Figure 2:**
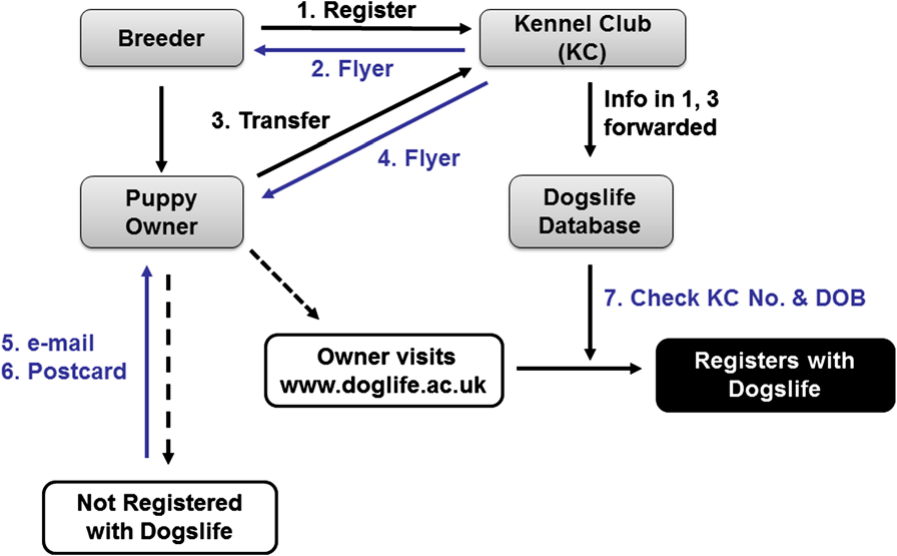
**Workflow description of recruitment into the Dogslife cohort. **The breeder registers a litter of puppies with the Kennel Club (KC) (1). A flyer advertising the project is included with the registration documentation provided by the KC (2). The puppy owner transfers registration of the puppy (3), and receives a flyer advertising the project with the transfer of registration documentation sent by the KC (4). The Dogslife database receives the KC number (KC No.) and date of birth (DOB) of all new puppy registrations (1) and transfers of registration (3). Puppy owners not registering with Dogslife are contacted by e-mail (5) and / or postcard (6) to encourage participation. The puppy owner joins the project and registers their puppy using the KC No. and DOB, which is checked by the Dogslife database (7).

The workflow for recruitment of dogs registered with the KC after the 1^st^ July 2010 is described in Figure 2. Delays with automation resulted in e-mails being sent from 12^th^ July 2010 and postcards from 9^th^ August 2010. After this time e-mail reminders were sent daily and postcards weekly.

To help launch the project and maximise recruitment, the project was actively publicised. The website was launched on the 1^st^ July 2010 with a press release published on the 30^th^ June 2010, which received national newspaper, television and press coverage. In January 2010 and January 2011 a short article detailing the aims of the project was sent to the secretaries of each Labrador Retriever breed club. Thirteen different Labrador Retriever breed clubs are listed on the Kennel Club website, and represent local or national groups with an interest in Labrador Retrievers. They were asked to disseminate it to their members through their yearbook or newsletter publication, and by adding information on the project to their breed club website. All breeders registering litters of Labrador Retriever puppies after the 1^st^ July 2010 were sent a black and white A5 flyer with their puppy registration documentation inviting them to join the project. Between July and September 2010 1,292 breeders of Labrador Retrievers listed on the Accredited Breeder page of the KC website, “Champdogs” (http://www.champdogs.co.uk), the Breeders Online (http://www.breedersonline.co.uk), Chocolate Labs (http://www.chocolatelabs.co.uk), Dog Club (http://www.dogclub.co.uk), K9 Puppy (http://www.k9puppy.co.uk) and UK Gundogs (http://www.ukgundogs.org) websites were contacted using the telephone number or e-mail address provided, to inform them about the project and to request their advocacy to future puppy owners. In December 2010 an article requesting further participation was forwarded to each of the breed clubs. In January 2011, 4,827 non-accredited KC registered breeders were contacted with a letter about the project and five copies of the postcard to distribute to new puppy owners.

### Website portal

Enrolment, routine and ad-hoc data entry was all performed by owners within a project website interface. The website design was subject to open tender in December 2009. The project was developed under the name “Dogslife” and the internet domain http://www.dogslife.ac.uk was secured from JANET, the UK’s education and research network. The website was registered with Google Analytics (http://www.google.com/analytics/) to enable real-time monitoring of the site usage metrics.

### Enrolment

All enrolments were a two-step process. The first required entry of the dog’s KC number, date of birth, age, gender and breed into a web page. If the KC number and date of birth matched those in the registration data supplied by the KC a second page requested an e-mail address and password. This process confirmed enrolment and entry to the dog’s “homepage”, where owners were requested to complete a single page questionnaire recording demographic information followed by a six-page questionnaire recording dog health and lifestyle information. The homepage also allowed owners to upload a photograph of their dog, visualise graphical representations of their dog’s data (height, weight, activity and illness episodes) and access links to the latest results from the study. Completion of the questionnaire could be deferred to the next site visit.

### Questionnaire

A web based questionnaire was designed and reviewed by epidemiologists, geneticists, veterinarians, and piloted with dog owners. A member of each Labrador breed club was invited by the KC to attend a focus group meeting in May 2010, where a draft version of the website was presented and subject to critique and open debate. The final version was pre-tested on dog-owners and amended accordingly.

The questionnaire requested information on the height and weight of the dog, the household environment, preventive healthcare measures, feeding, exercise, intended use, and health or illness. Data was collected in categorical, textural and nominal format and took a maximum of 10 minutes to complete. All questions on each page were compulsory (i.e. required answering for the website to allow progress onto the next page) with the exception of weight (which owners could leave blank). Weight data was not compulsory as it was perceived that some owners may have difficulty in obtaining this measure. When owners left the questionnaire section of the website before answering all of the questions, and did not return to complete the information within the data entry window (see below) the questionnaire was recorded as being “incomplete”. A paper version of the questionnaire was not offered due to the cost and logistical reasons.

### Compliance and retention

The owners of participating dogs were requested to complete the web-based questionnaire about their dog’s health and welfare at 30 day intervals for the first year of the dog’s life and at three monthly intervals thereafter. Reminders for non-completed monthly questionnaires were sent by automated e-mail one week after the deadline (30 + 7 days) and by telephone or non-automated e-mail two weeks after the deadline (30 + 14 days). If the questionnaire was still not completed, further reminders were sent 84 (automated e-mail) and 91 days (telephone or non-automated e-mail contact) after the date of the last completed data entry. If no response was received after 91 days the dog was listed as lapsed. All contacts by telephone or non-automated e-mail were made on the date, or as close after the date as possible (for example if the contact date was at a weekend, the contact was made on the following Monday). For entries up to the first year of age, the reminder timeline was reset after the data entry. For example, if the owner joined the project and completed their first data entry when their dog was 90 days old, and was due to complete the next data entry 30 days later, when their dog was 120 days old, but did not complete it until their dog was 130 days old (10 days after the data entry was expected (120 days) and three days after a reminder e-mail was sent (120 days +7 days =127 days)) then the following data entry was expected 30 days later when their dog was 160 days old (130 days + 30 days), and if this did not occur, the next e-mail reminder would occur at 167 days (130 days + 30 days + 7 days). However, there was considerable ‘slippage’ in the interval between data entries, so after one year of age when the response frequency dropped to every three months, a data entry window was established to facilitate responses and control this problem. This extended from 21 days before to 35 days after each of 3 monthly expected data entry dates. Non-responders were sent an e-mail reminder at 7 days after the expected date and contacted by telephone or non-automated e-mail 14 days after the expected date. Owners could also enter data outside these timelines, and as frequently as they wished.

To encourage retention, a monthly newsletter containing articles about canine health, Labrador Retrievers and project updates was started in August 2010. The newsletter was e-mailed to all participants wishing to receive it, and to members of the general public who had registered an e-mail address with the website.

A three monthly prize draw was instigated for the first twelve months of the study to incentivise enrolment and retention. Each complete data entry was accorded one entry in the draw, with thirteen prizes to the value of £100 (one), £50 (two) and £10 (ten) awarded. The Dogslife website hosted an editable scrapbook with the facility to upload photographs and input text detailing memorable events. Owners had the opportunity to nominate their pet as “Featured Dog”. A picture and description of these dogs appeared on the website home-page.

### Illness data

In the last section of the questionnaire, owners were asked to report any illness their dog(s) suffered. This section could also be accessed at any time point. Owners classified their dog’s illness into one or more of six broad clinical syndromes: vomiting, diarrhoea, scratching, licking and chewing themselves, coughing, and lameness. An open question also allowed the owner to describe any illnesses or clinical signs not listed. When an illness was reported, participants were asked to detail the duration, frequency, veterinary visits, and treatment.

If the owner made a visit to their veterinary surgeon, they were asked in addition to complete a Dogslife Health Record form or ask the veterinary surgeon to do so for them. This was a single page form available from the website which enabled details of the clinical signs, diagnosis and treatment of non-routine veterinary presentations to be recorded. Owners were encouraged to print the form, keep it with their pet’s vaccination reminder, and ask the veterinarian to complete it at non-routine presentation. They were then asked to transfer this information to the on-line questionnaire. The presenting signs and diagnosis were coded using the VeNom standard veterinary nomenclature code list [20] by an experienced clinician (DNC). Ambiguities in clinical signs or diagnosis were resolved by contacting the owner by telephone or e-mail.

### Data analysis

**Figure 3:**
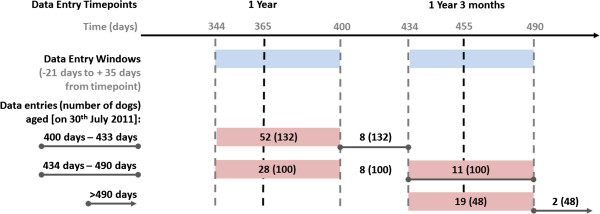
**A profile of the data entry windows where participants were considered “active” for dogs aged 400 days or older on 30**^**th **^**June 2011. **The number of dogs with a complete data entry is listed with the total number of dogs eligible for a data entry in the window in brackets.

Retention was estimated using the 280 dogs that were at least 400 days of age on the 30^th^ June 2011. This age was selected on the basis that this would encompass the first data entry window for all dogs who had reached one year of age (344 to 400 days). As some dogs would have been old enough to reach the second data window (at 1 year 3 months of age, with the window extending from 434 to 490 days of age) retention was defined as those dogs which had a complete data entry in the last data entry “window” where a data entry was expected (Figure 3). Participants were considered active:

1. If the dog was less than 434 days of age and a data entry was recorded in the 1 year of age window (between 344 and 400 days of age),

2. If the dog was between 434 and 490 days of age and a data entry was recorded in either the 1 year of age window (between 344 and 400 days of age) or the 1 year 3 months of age window (between 434 and 490 days),

3. If the dog was older than 490 days of age and a data entry was recorded in the 1 year of age window (between 434 and 490 days).

The number of participants entering data outside the requested windows (between 401 and 433 days of age and after 490 days of age) was also recorded.

The geographical distribution of recruited dogs was determined by using the first two letters of the postal code which localised the dog to a postal delivery area. The proportion of dogs recruited from each area was estimated from the number of dogs recruited from each postcode divided by the total number of Labrador Retrievers reported by the KC to be from that postcode. Dogs missing postcodes were removed. The maps were produced using the R software environment [21].

The time to the first owner-reported illness and the time to the first non-routine veterinary presentation were estimated using standard Kaplan-Meier survival model with 95% confidence intervals (using the R 'survival' package [22]). The data were naturally left truncated as dogs were not enrolled in the study from birth and were right censored at their last data entry.

### Withdrawal from the study

Participants were able to withdraw from the study at any point by e-mailing the study team. For members leaving the study because they no longer had their dog the reasons for exit from the study were requested in a free text field.

## Results

### Recruitment

**Figure 4:**
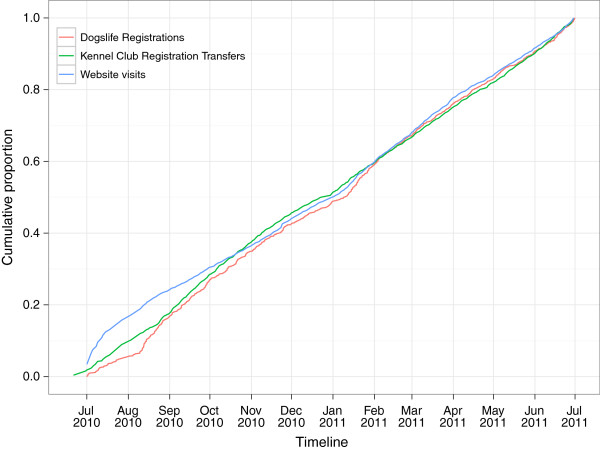
**Profiles of the timescale between recruitment and active intervention. **The cumulative proportion of dogs registered (red line), dogs actively targeted (Labrador Retrievers whose registration has been transferred with the Kennel Club (KC), green line) and visits to the website (http://www.dogslife.ac.uk, blue line) are presented over the first year of the project. The number of website visits on the first day of the project (1^st ^July 2010) was high, hence the cumulative proportion on this date appears to be greater than 0. The KC registration transfers also started in advance of 1^st ^July 2010. The e-mail and postcard reminders are initiated 7 and 14 days after the transfer of registration.

Between 1^st^ July 2010 and 30th June 2011 1,407 Labrador Retrievers (from 1,384 owners) were enrolled in the study. Overall this represents 2.4% of the 58,735 eligible dogs. The mean rate of recruitment was 117 dogs (standard deviation [SD] 24 dogs) per month, and apart from some fluctuation in the first three months of recruitment, it remained fairly constant through the first year of the project (Figure 4). Again, with the exception of the first month, the recruitment rate closely followed both the transfers of registration and the number of new website visits.

**Figure 5:**
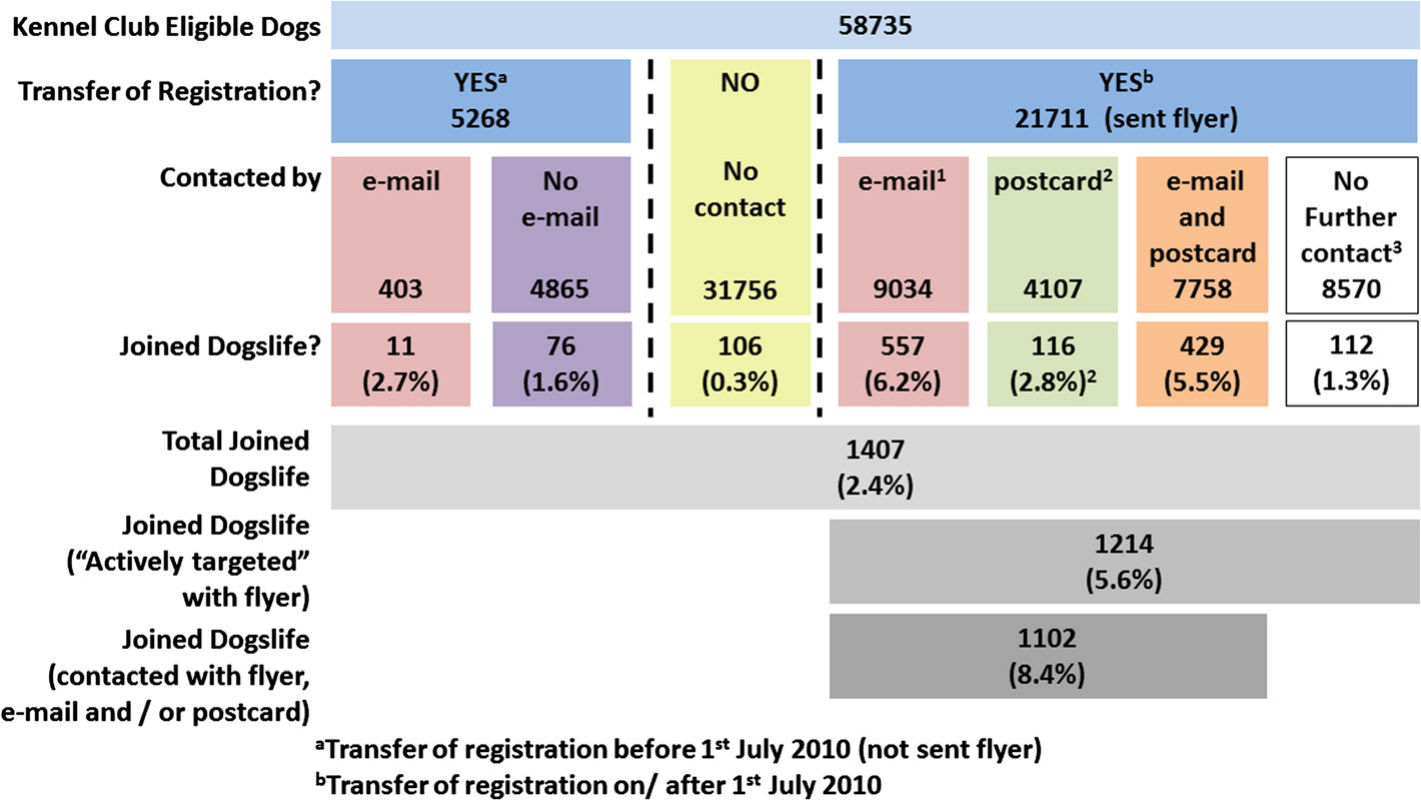
**Pattern of recruitment by different methods. **After 1^st ^July 2010 all transfers of registration were sent a flyer advertising the project with their transfer documentation. ^1^The number of people contacted by e-mail includes those who were subsequently contacted by postcard when they did not join the project. ^2^The number of people contacted by mail excludes those who were also previously contacted by postcard. ^3^No contact by e-mail or post includes 528 dogs with e-mail and postal addresses, but who had not been sent the contact by 30^th ^June 2011 as the timeline for contact (7 and 14 days after transfer of KC registration respectively) had not elapsed.

**Figure 6:**
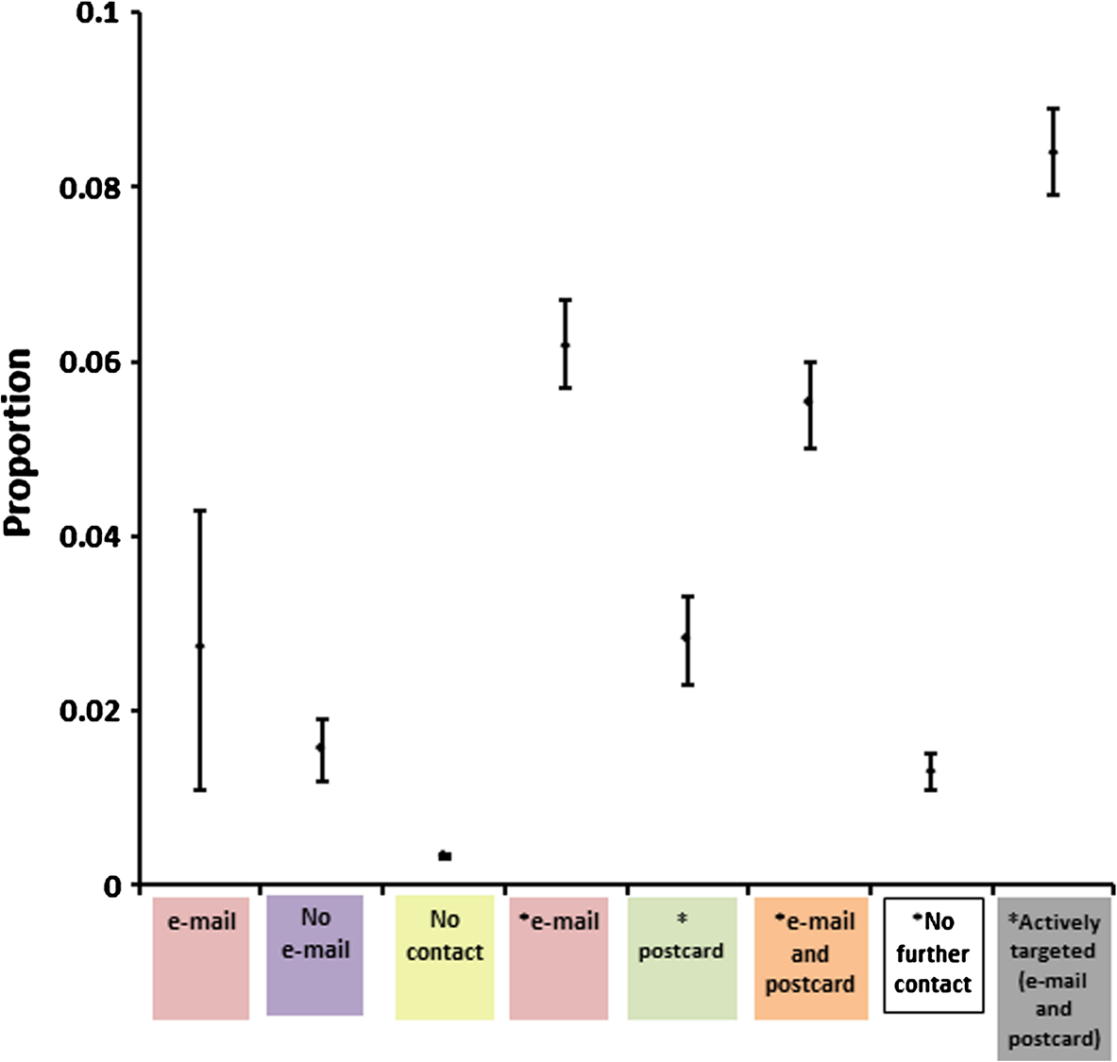
Proportion of eligible dogs recruited by different methods with 95% confidence intervals. *dogs with transfer of registration on or after 1^st ^July 2010.

**Figure 7:**
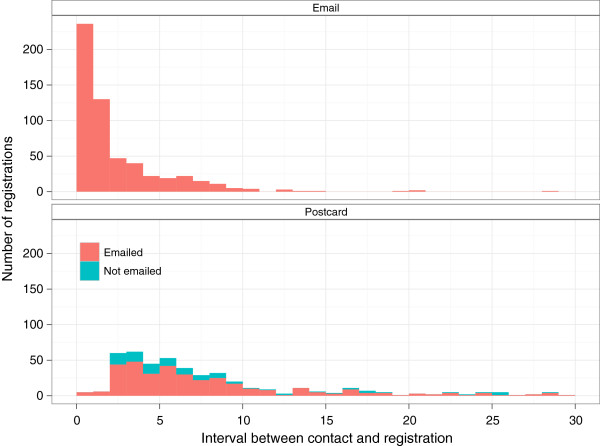
**Profiles of the timescale between recruitment and active intervention. **The profile of the number of responses to the postcard shows that the majority had previously received, but not responded to, an e-mail requesting participation in the project.

Of the 21,711 eligible dogs recorded as having a transfer of registration with the KC between July 1^st^ 2010 and 30^th^ June 2011, contact details (postal address and e-mail address) for owners were provided for 60.5% (13,141). 1,102 of these dogs were recruited giving an active recruitment rate of 8.4% (Figure 5). Contact by automated e-mail and by postal mail was associated with an increased recruitment of transferred dogs. A single e-mail message was associated with an increase in recruitment from 1.3% to 6.2% and when followed by a postal reminder this increased to 8.4% (Figure 5). A single postal reminder was apparently less effective, being associated with a recruitment increase from 1.3% to 2.8%. Overall 54.1% (31,756) of dogs did not have their registration transferred from the breeder to a new owner. New owners in this group received no direct information about the project but surprisingly a small percentage (0.3%, 106) enrolled into the Dogslife cohort. The proportion of participants recruited where a transfer of registration was reported (and hence they received the initial flyer), without any further e-mail or postal contact, was similar throughout the project at 1.3-1.6% (Figure 6). The proportion of dogs recruited after e-mail contact was higher when preceded with a flyer (6.2%) at the transfer of registration, and the e-mail being sent 7 days after the transfer of registration, than where no flyer was sent (2.7%) (Figure 6). The response rate after an e-mail reminder was rapid, usually occurring within 48 hours of the e-mail being sent. In contrast, response after the postal reminder was slower with the majority of new registrations occurring within two weeks after the postcard was sent (Figure 7). When asked by telephone 117 of 677 breeders (17.5%) reported that they were aware of the project, and 488 (72.1%) were supportive of the project.

### Compliance and retention

Of the 1,407 dogs enrolled with the project on the 30^th^ June 2011, 907 dogs (64.4%) had a complete first data entry and 500 (35.6%) dogs were recorded as having a partial first data entry (36.0%). There were 4,422 data entries in the first year of the study, of which 3,413 (77.2%) were complete. Details of the “drop out point” (the last page of the questionnaire reached in the partial data entries) are shown in Table 1, and identify that 77.6% (383) of the partial entries on the first visit to the site were terminated at the demographic, and weight and height pages. Overall the weight and height page was the point at which the vast majority of data entries (50.6%) were prematurely terminated when subsequent visits were also included.

**Table 1:** Number of complete and partial data entries at the first visit to the website and at subsequent visits

	**First visit**	**All visits**
Full Data Entry	907 (64.4%)	3413 (77.2%)
Partial Data Entry	500 (35.6%)	1009 (22.9%)
Household Demographics*	190 (38.0%)	190 (18.8%)
*Height and weight (Page 1)*	193 (38.6%)	511 (50.6%)
*Bathing / Veterinary Care (Page 2)*	59 (11.8%)	156 (15.5%)
*Exercise (Page 3)*	10 (2.0%)	31 (3.1%)
*Feeding (Page 4)*	16 (3.2%)	32 (3.2%)
*Routine Healthcare (Page 5)*	18 (3.6%)	40 (4.0%)
*Illness (Page 6)*	14 (2.8%)	49 (4.9%)

For partial data entries the last completed web-page of the questionnaire is detailed in italics with the percentage of partial enrolments that terminated at that page. *Information about the household demographics was only asked for at the first data entry.

**Figure 8:**
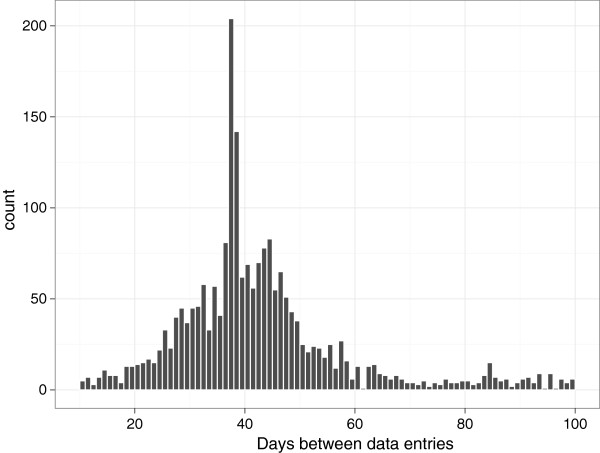
A profile of the timescale between completed data entries in the cohort.

There was considerable variation in the frequency of data entry reflected in the wide range of intervals between data entries. The aim was to collect data every 30 days. However the intervals varied from 10 to 100 days with peaks at 37 days and 45 days, which corresponded with the timing of the e-mail and the telephone reminders respectively (Figure 8).

Thirty nine per cent (110) of the 280 dogs aged 400 days or older on the 30^th^ June 2011 were considered to be “actively” participating in the project. A further 6% (18) had a completed data entry after the last data entry window in which they were requested to enter data, but not within the data windows requested (Figure 3).

Overall twelve dogs were withdrawn from the study by 30^th^ June 2010; one dog died, two dogs were put to sleep (one with severe hip dysplasia; the aetiology of the second case was not reported), two dogs were rehomed for behavioural problems, and seven were rehomed without the reason reported.

### Signalment of participants

The distribution of coat colours of dogs enrolled in the cohort was: black 691 (49.2%, 95% Confidence intervals [CI] 46.5%, 51.7%), yellow 367 (26.1%, 95% CI 23.8%, 28.4%), chocolate 314 (22.4% 95% CI 20.2%, 24.5%), other 32 (2.3%, 95% CI 1.5%, 3.1%). Coat colour was not recorded in three dogs. In comparison the distribution of coat colours of eligible dogs was: black 30,630 (52.2%, 95% CI 51.7%, 52.6%), yellow 16,850 (28.7%, 95% CI 28.3%, 29.1%), chocolate 11,157 (19.0%, 95% CI 18.7%, 19.3%), other 98 (0.17%, 95% CI 0.13%, 0.20%). The number of males and females registered was roughly equal (721 (51.3%, 95% CI 48.7%, 51.9%) males, 686 (48.7%, 95% CI 46.1%, 50.3%) females), which was similar to that of the eligible population of 29,464 (50.2%, 95% CI 49.8%, 50.6%) males, 29,271 (49.8%, 95% CI 49.4%, 50.2%) females.

**Figure 9:**
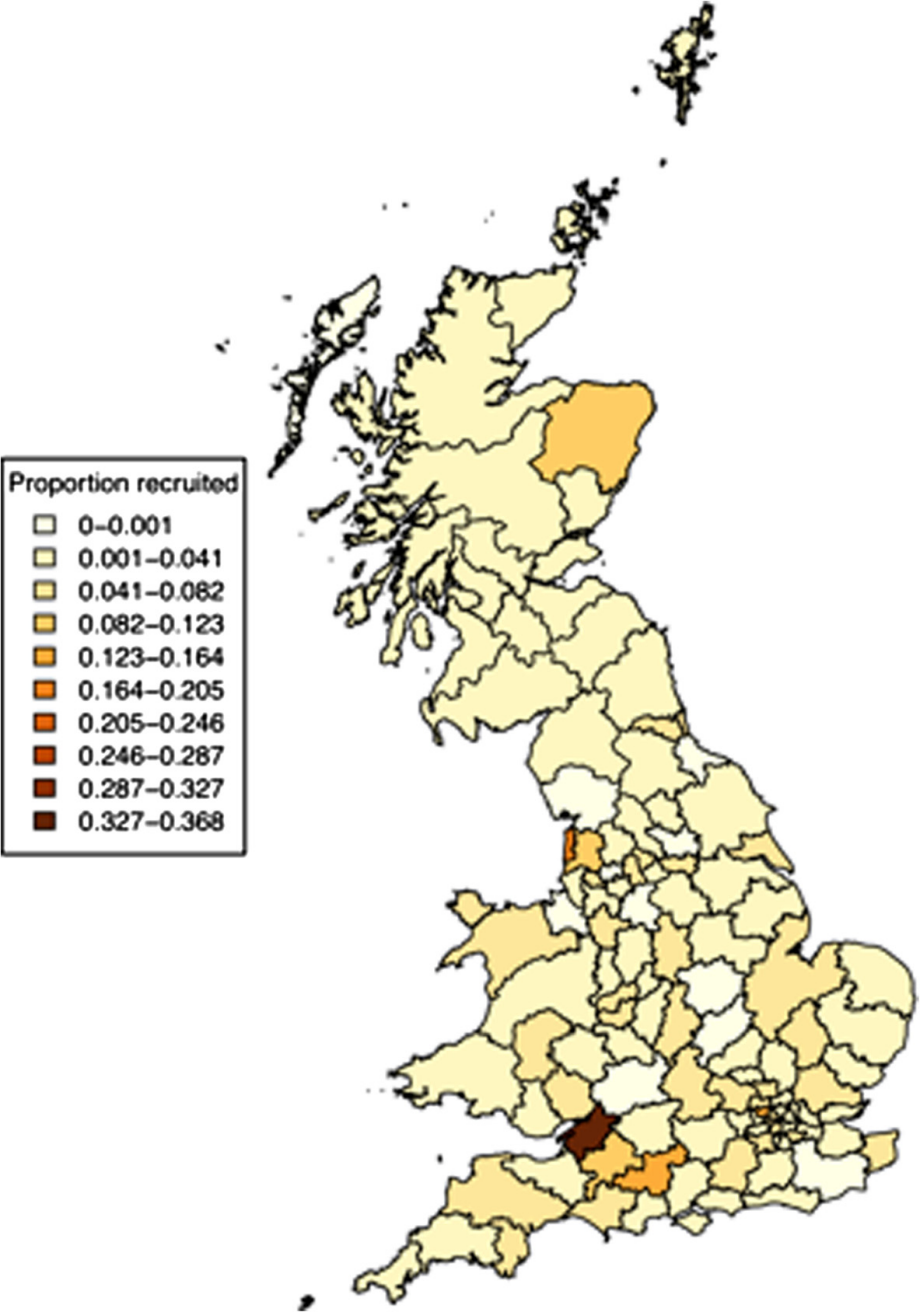
**The geographic distribution of the proportions of eligible participants registering with the Dogslife project. **Location was defined by the first two letters of the postcode forwarded from the Kennel Club, and the proportion of eligible dogs registering with the study is highlighted on the right hand scale. The graph scales across 7 standard deviations (<−1SD [0.000-0.003], -1SD – 0SD [0.003-0.041], 0SD-1SD [0.041-0.082], 1SD-2SD [0.082-0.123], 2SD-3SD [0.123-0.164], 3SD-4SD [0.164-0.205], 4SD-5SD [0.205-0.246], 5SD-6SD [0.246-0.287], 6SD-7SD [0.287-0.3327]), 7SD-8SD [0.327-0.368]).

The relative proportion of eligible dogs registered with the KC who were enrolled in the project by postal region (as defined by the first two letters in their postcode) is shown in Figure 9. The proportion of members joining was relatively evenly distributed across the 122 postal code areas, with the exception of Bristol where a higher proportion of potential participants joined.

### Demographics

1,372 owners registered a single dog, 12 owners registered two dogs, two owners registered three dogs and one owner registered five dogs. The type of households owning dogs was reported as: 45.7% (627) “Family” (one or more adults and one or more children), 39.9% (548) “More than one adult and no children”, 6.0% (82) “Single or couple retired”, 5.7% (78) “Single adult”, 0.6% (7) “Other household type” and no response was given by 34 (2.5%). 235 (17.1%) of households joining the project reportedly contained a smoker. 1,323 (96.1%) of households contained another pet. Most commonly this was another dog (46.0%) or a cat (29.7%), a combination of other dogs and cats (8.2%), or other species with or without dogs and/or cats (12.2%).

### Health

**Figure 10:**
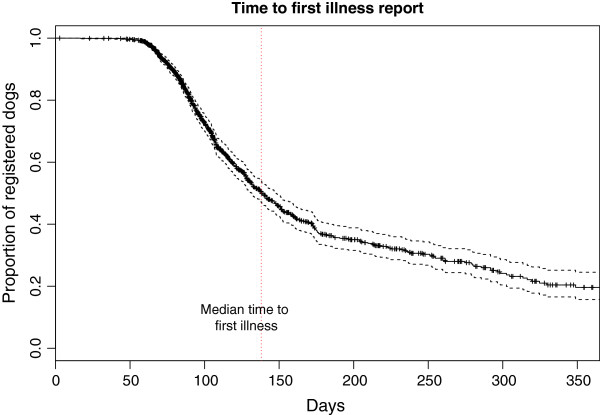
Survival analysis of the time to first owner-reported illness with 95% confidence intervals.

**Figure 11:**
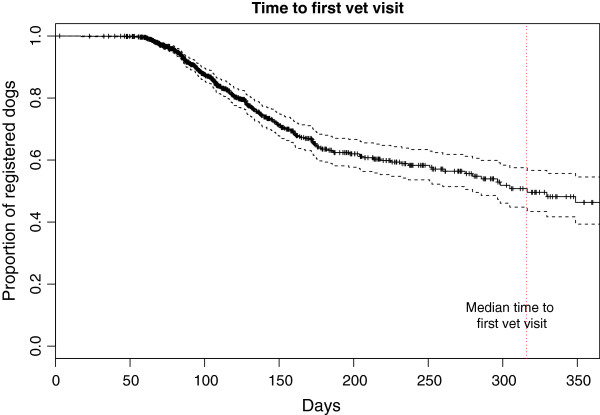
Survival analysis of the time to first non-routine veterinary presentation (B) with 95% confidence intervals.

Clinical signs or illnesses were reported in 44.3% of the cohort (median age 138 days, 95% CI 132 days - 148 days) and in 51.7% of dogs aged 1 year or older on the 30^th^ June 2011. When corrected for censoring (analysing dogs only over the period of their data entry/entries) by survival analysis, 80.4% (95% CI 75.5% - 84.3%) of dogs were estimated to have developed an illness by 1 year of age (Figure 10). Of the reported illness “episodes” 44.1% were taken for veterinary attention (median age 316 days, 95% CI 280 days – no upper limit), with 35.1% of dogs aged 1 year or older on the 30^th^ of June 2011 being presented for non-routine veterinary attention. When corrected for censoring by survival analysis, 53.7% (95% CI 45.4% - 60.7%) of dogs were estimated to have presented to veterinarian for non-routine attention by one year of age (Figure 11). This indicates that the many of clinical signs reported by owners were perceived to be mild or resolved without the requirement for veterinary attention.

### Website metrics

The website received 21,771 visits, from 8,047 unique visitors, between 1^st^ July 2010 and 30^th^ June 2011. Mobile devices were used to access the site in 7.1% (1,549) of visits. The majority of unique visitors arrived through direct entry of the domain name into their internet browser or clicking the link imbedded in the e-mail (40.6%) or by searching for the domain on Google (16.6%) (Table 2). The health links page was viewed 892 times by 791 unique visitors, with 356 visitors exiting via one of the links. The featured dog archive was viewed 1,739 times by 1,368 unique visitors, although the number of visitors viewing the featured dog pop-up on the home page could not be calculated, as it was not recorded in the metrics. 149 dogs were nominated as “featured dogs” in the first year of the project.

**Table 2:** **Source of new website traffic to http://www.dogslife.ac.uk**

**Source**	**Number of visitors**	**Percentage**
Direct	3265	40.6
google	1337	16.6
the-kennel-club.org.uk	411	5.1
ed.ac.uk	292	3.6
labradorforums.co.uk	239	3
news.bbc.co.uk	222	2.8
thekennelclub.org.uk	163	2
facebook.com	151	1.9
google.co.uk	117	1.5
uk.mg.bt.mail.yahoo.com	116	1.4
Other	1734	21.5
**Total**	8047	100

8047 unique visits, between 1st July 2010 and 30th June 2011.

### Motivation for participation

Of the 149 dogs nominated as “featured dogs” all owners reported one or more reasons for joining the project. They were: to help with research (104 owners, 71%), to compare or monitor their own dog’s health, progress or development (40 owners, 27%), or other reasons (usually for interest or further information, 22 owners, 15%).

### Newsletter and scrapbook

91% (1262 participants) requested the newsletter. An additional 152 non-participants also requested to be included in the newsletter distribution list. The scrapbook feature was taken up by 47% of participants (652), and the scrapbook page was viewed 3,724 times.

## Discussion

### Recruitment

Recruitment and retention of a representative population, and compliance and the collection of accurate data are the four cornerstones of longitudinal studies. Recruitment of a representative population is a major challenge for all longitudinal studies. This is commonly attempted by targeting a birth cohort restricted in either time or location, but it is rarely achieved as participation will be influenced by many factors. Assessment of the representativeness of the recruited population is usually achieved by comparison to reference values collected in the general population such as demographics or weight [23], or by comparison to other measures such as disease incidence in other similar cohorts [24]. In production animals this has been achieved by studying individual herds and flocks [12-14]. In pets it presents a major problem because in most countries there is no national registration system for all animals and so the demographics of the general population are unknown. To overcome these difficulties we chose one breed of UK Kennel Club registered pedigree animals. The Labrador Retriever breed was chosen as it is the most common pedigree dog in the UK. They are bred by individuals and registration of their dogs with the KC confers certified pedigree status on these animals.

The overall response rate (2.4%) was very similar to that reported for a human internet-based birth cohort study (3.7%) [25]. Although it appears limited, a large proportion of the eligible population were not actively targeted either because the owner did not transfer the dog’s registration with the KC or did not consent to be contacted by post and/or email. Thus 71% of eligible dogs were not actively recruited. When targeted with flyers, e-mail and postcards, then the recruitment rate increased to 8.4%. Offering a paper-based alternative questionnaire may have increased the recruitment [17,26-28] and retention rates [17]. The use of repeated e-mail and postal reminders may also have increased the recruitment rate further [17]. However a significant cost implication is associated with using postal services; the limited budget of this project restricted postal communications to a single invitation to each potential new member (where we had permission to do so). We did not request owners to detail where they heard about the project to support the inferences we have made based on the time of enrolment relative to the recruitment timeline.

The relatively low “conversion rate” of website visitors to participants (17.6%) suggests that although many people found the study intriguing enough to view the website, they did not necessarily go on to register. The relatively high frequency of data input (monthly until one year of age) may also have been a deterrent to potential participants. Although not specifically studied, informal verbal feedback from non-participants indicated that the time required to participate in the study was the primary reason for non- participation, which is consistent with reasons given in other longitudinal studies [29]. Clearly, follow up of a cohort of non-participants is required to determine the representativeness of the cohort, although comparison with the geographical, coat-colour and gender data available from the population of dogs eligible to register did not identify any overt differences.

Designing and populating a website to appeal to a section of the dog-owning public was challenging. The spectrum of households owning a dog in the UK is broad, although dog ownership is strongly associated with families who have children [30], and this was supported by the household types reported by participants in the project. Consequently the colour pallet and graphics of the website were designed to be relatively neutral to appeal to all age groups whom might use the site. We used many of the recommendations known to increase responses to paper and electronic questionnaires, namely the assurance of confidentiality, the use of non-monetary incentive, the notification of results, using a university affiliation, designing the questionnaire with simple headers and a white background and giving textural representation of response categories [31]. We also obtained an “academic” domain name (ending .ac.uk) to validate the authenticity of the project. Over half of new visitors found the website directly or via a Google search, which implies that they were directed to the website by the flyer, reminder e-mail or postal contact. This was also supported by the observation that the registration trend line is relatively linear throughout the year, rather than demonstrating an exponential increase in activity, which might have been expected if the project had gained awareness or popularity through other means. Although social media sites, such as Twitter and Facebook, can be used to recruit new participants to longitudinal studies [32], we avoided this form of communication in the first year of the project as we wished to maintain the independence of participants and avoid the potential influence of inter-participant communication through the network.

### Compliance and retention

One of the findings of our analysis was that the timelines for data entry were generally not adhered to, and “questionnaire fatigue” was undoubtedly a factor in participant loss and the low compliance with the data entry timelines. The aim was to collect data every 30 days but the data entry intervals were between 10 and 100 days.

The compliance with entering the requested data to the questionnaire was reasonably high, with nearly 80% of data entries being complete. Unsurprisingly the proportion of complete data entries increased after the first data entry, as participants completing their first data entry would be expected to be more likely to complete subsequent entries. Partial data entries occurred with members stopping data entry at any point of the questionnaire, although the vast majority stopped on the first two web-pages of the questionnaire. The first web-page required owners to have measured the dogs’ height and weight, and the second requested information about bathing their dog. Progress from one web-page to the next was conditional on the owner having completed the answers to the questions on the previous page (with the exception of the weight of the dog). The subsequent four web-pages were completed in the majority of cases, with roughly 1% of data entries being stopped at each page, although the number of questions on the subsequent web-pages (exercise, feeding, routine healthcare and illness) was greater than on the second page. This supports comments made to the project secretary during telephone conversations with participants, that members found obtaining the physical measurements onerous, and the repetitiveness of questionnaire tiresome. It is possible that a memo on the first page of the questionnaire reminding participants of the need for these measures, reordering these questions or pre-populating data entries with the previous results, might have increased the proportion of complete data entries. Clearly, if owners had gone to the trouble of taking the measures of height and weight they were likely to persist with the remaining data entry into the questionnaire.

Owners of 39% of dogs were estimated to be actively involved in the study after their dog had reached 400 days of age, and a further 6% provided data entries outside the requested guidelines. This rate is less than that reported for other internet based longitudinal studies of human health (60-72%) [33], and a conventional longitudinal study of health utilising internet, telephone and paper-based communication (79%) [34]. Considering the relatively high commitment by participants of the study, both in the number of measures taken and frequency of data entry, and the absence of direct reward to participants, the retention rate was not surprising. We used wide “windows” to calculate data retention, as it was felt unnecessary to request re-entry of data less than 21 days from the optimal data entry timelines (1 year of age, and 1 year 3 months of age). Similarly, we arbitrarily closed the data entry 21 days after the second reminder (telephone), because the profile of data entries showed that many participants would take up to 3 weeks after a phone call to enter data into the website. The peaks in data entry frequency around the date of reminders showed the value of these in encouraging punctuality. However, participants who did not respond to reminders at 37 and 44 days did not commonly respond to the reminders at 84 and 91 days, suggesting this second reminder timeline was not particularly beneficial. It is possible that the time interval between the second and third reminders was too great and that more frequent reminders around the end of each 30 day period may be more satisfactory in enhancing retention.

The motivation to participate in the study was primarily altruism, based on information supplied to the “featured dog” section and informal discussions with participants during telephone reminders. For the majority of participants in the “featured dog” section it was the desire to help research into their dog’s breed, rather than the personal benefits of the record keeping or desire to find information, that was stated as the reason for joining the project. This was also indicated by the observation that the fun features of the website such as the “featured dog” and scrapbook pages were more popular than the health information links.

### Population

The colour and gender data of the cohort suggest that Dogslife is broadly representative of the general Labrador Retriever population. The slight increase in the number of chocolate and other coat coloured dogs in the Dogslife cohort when compared to the general Labrador Retriever population was not perceived to intimate an important bias in the representativeness of the cohort. A relatively low prevalence of smoking (17%) was recorded in the owner cohort compared to that reported for the adult population in England and Wales (21% [35]) or Scotland (24% [36]). It is well recognised that both adults and children owning dogs are more physically active [37,38], and thus might be less likely to smoke. Smokers are also more likely to be of low socioeconomic status [39] and hence less able to afford to purchase and maintain a large pedigree dog.

The frequency of clinical signs or illnesses reported in the first year of life was high, although only a proportion resulting in veterinary visits, suggesting that many were regarded as being of low severity or unimportant. The estimated frequency of non-routine veterinary presentations (54%) in the first year of life of dogs participating in the project was much higher than the reported annual risk of making a veterinary care insurance claim from the age of two onwards [40], and is an underestimate as most of the cohort had not reached one year of age. Young children also show an increase in the number of primary healthcare presentations per annum in comparison to other age groups, although the rate we recorded in the Dogslife cohort was still lower than the comparative values for children (a mean of 6 general practitioner consultations per person-year in children aged 0–4 years) [41].

One of the major challenges for the study was to quantify the validity and reliability of the clinical data provided. We anticipated that the Health Record form would provide a means of expediting transfer of this data, but its usage appeared limited. Integration with computerised health records for veterinary epidemiological studies has been performed [42], but the plethora of different record management systems used and the requirement to obtain agreement from participating veterinarians make this unachievable in the short term in a nationwide study such as Dogslife. Consequently, recall of veterinary health records of a random selection of the cohort is being undertaken to ascertain the accuracy and completeness of the data recorded to date.

Although difficult to quantify, we believe that secretarial support was fundamental to the recruitment and retention process, in facilitating the postal contact, telephone reminders, answering queries, troubleshooting website problems and providing a point of human contact to an otherwise computerised process.

## Conclusions

The Dogslife project has demonstrated the utility and problems associated with internet-based longitudinal observational studies. Recruitment to such studies can be enhanced additively by e-mail or postcard contact with the target population. We believe Dogslife will provide a useful insight into the demographics of Labrador Retriever ownership and health status through the recording of health information and will allow us to investigate a wide range of environmental influences on reported illnesses in the future.

## Abbreviations

KC: Kennel Club; UK: United Kingdom.

## Competing interests

None of the authors have any competing interests which would influence the results of this study.

## Authors’ contributions

DNC, WERO, LK, KLM, BMB, IGH, KMS, DQ and JS conceived the study; DNC, BMB, IGH, KMS and DQ designed, tested and refined the website and questionnaire; DNC, ER and CAP collected the data; DNC, IGH, KLM, DQ, KMS, BMB and CAP analysed and interpreted the data; all authors read, contributed to and approved the final manuscript.

## Authors’ information

Kim M Summers and B Mark C de Bronsvoort are the joint last authors.
